# GPRIN1 enhances gallbladder cancer progression and is associated with the CDK1 pathway

**DOI:** 10.1080/15384047.2026.2753819

**Published:** 2026-11-05

**Authors:** Min Li, Xiaojian Ni, Zijun Gong, Lujun Song, Tao Suo, Han Liu, Sheng Shen, Houbao Liu, Xiaoling Ni

**Affiliations:** a Department of General Surgery & Biliary Surgery, Zhongshan Hospital, Fudan University, Shanghai, China; b Shanghai Engineering Research Center of Biliary Tract Minimal Invasive Surgery and Materials, Shanghai, China; c Biliary Tract Disease Institute, Fudan University, Shanghai, China

**Keywords:** Gallbladder neoplasms, GPRIN1, phosphoproteins, proteome, cyclin-dependent kinases

## Abstract

G protein-regulated inducer of neurite outgrowth 1 (GPRIN1), a predominantly membrane-bound protein highly enriched in neurite growth cones and neurite outgrowth, plays a crucial role in neuronal network development. Recent studies have also shown that GPRIN1 participates in regulating cancer proliferation and migration. However, there is limited research regarding its role in gallbladder cancer (GBC). In this study, we found that GPRIN1 expression was upregulated in GBC tissues (*p* < 0.01) compared to normal tissues, and high GPRIN1 expression was positively correlated with poor prognosis (*p* < 0.001) in GBC patients. Both in vitro and in vivo studies showed that high GPRIN1 expression reinforced the proliferative and invasive capacities of GBC. Coimmunoprecipitation assays and mass spectrometry were performed to identify GPRIN1-interacting proteins. Biosynthesis analysis and Western blot further validated that there was a direct interaction between GPRIN1 and cyclin-dependent protein kinase 1 (CDK1). Then, cell proliferation, MTT and Transwell assays were performed to verify the functional recovery effect of CDK1 on GPRIN1. Moreover, we performed proteomic analysis of differentially expressed proteins and phosphorylated peptides separately, and a combined analysis was subsequently performed, which revealed the involvement of CDK1 in many biological processes and cellular pathways. Collectively, GPRIN1 enhances GBC cell invasion and metastasis, potentially through activation of the CDK1 pathway.

## Introduction

Gallbladder cancer (GBC) is the most common type of biliary tract cancer worldwide and the fifth most common gastrointestinal malignancy in Western countries.^1^ As a potentially lethal disease, GBC is associated with an extremely poor prognosis, with a 5-y survival rate of 5%–18%, because of its high propensity for invasion and metastasis.^2^ Only 15%–47% of newly diagnosed GBC patients in an early stage can be treated with surgery, and most GBC patients are not candidates for surgery because of early metastasis.^3^ Currently, complete surgical resection remains the only potentially curative treatment for GBC.^4^ Due to the anatomical characteristics of the gallbladder itself, GBC can spread through adjacent liver metastasis, lymph node metastasis, vascular metastasis, neural metastasis, intraperitoneal metastasis, and intraductal metastasis1.^1^ Therefore, identifying the mechanisms that mediate GBC invasion and metastasis is essential for improving clinical treatment strategies for GBC. However, the underlying molecular mechanisms involving GBC metastasis remain poorly understood.

G protein regulated inducer of neurite outgrowth 1 (GPRIN1), a membrane-bound protein enriched in neurite growth cones, selectively binds activated Gαo, Gαz, and Gαi to promote neurite outgrowth5.^5^ ^,^ ^6^ Beyond its established function in neuronal network formation, GPRIN1 has been identified as an independent prognostic biomarker for lung adenocarcinoma, demonstrating regulatory control over cancer cell proliferation and metastatic potential.^7^ Pan-cancer analyses of TCGA (The Cancer Genome Atlas) datasets further reveal significant GPRIN1 upregulation across multiple malignancies, suggesting its broad involvement in tumorigenesis.^8^ ^,^ ^9^ While these collective findings underscore GPRIN1's critical contributions to tumor progression and metastasis, the precise molecular mechanisms governing its oncogenic activity remain elusive. Notably, the functional characterization of GPRIN1 in GBC represents a critical knowledge gap, with no systematic investigations reported to date.

In the present study, we found that GPRIN1 was expressed at a higher level in GBC tumor tissues, and high GPRIN1 expression was positively correlated with poor prognosis. We found that GPRIN1 promoted the proliferation, migration and invasion of GBC cells in vitro. Additionally, in vivo experiments confirmed that high GPRIN1 expression reinforced the proliferative and invasive capacities of GBC. Coimmunoprecipitation (co-IP) assays and mass spectrometry (MS) were performed to identify GPRIN1-interacting proteins. Gene products that may interact with GPRINI were chosen on the basis of a biosynthesis analysis and verified by Western blotting, which showed a direct interaction between GPRIN1 and cyclin-dependent protein kinase 1 (CDK1). CDK1, which is conserved in all organisms and plays important roles in mitosis and drives cells into the S phase without CDK2, has been shown in recent studies to be overexpressed and valuable for estimating the prognosis of various cancers.^10^ ^,^ ^11^ Cell proliferation, MTT and Transwell assays suggested that CDK1 exerted a functional recovery effect on GPRIN1. Moreover, we performed proteomic analysis of differentially expressed proteins and phosphorylated peptides separately, which revealed the involvement of CDK1 in many biological processes and cellular pathways. In conclusion, our study demonstrates that GPRIN1 promotes gallbladder cancer progression, likely through functional interaction with the CDK1 signaling pathway. These findings suggest that therapeutic targeting of this axis—either through GPRIN1 inhibition or CDK1-directed agents—may offer a promising therapeutic strategy for GBC, warranting further preclinical investigation.

## Results

### GPRIN1 expression is correlated with clinical pathological features and prognosis in patients with GBC

Immunohistochemistry analysis revealed the upregulated expression of GPRIN1 in GBC (Figure 1A). And GPRIN1 expression in GBC was significantly higher than that in normal gallbladder tissues (*p* < 0.01, Figure 1B). Moreover, the relationship between clinical pathological characteristics and GPRIN1 expression is shown in Table 1, which indicated that overexpression of GPRIN1 in GBC was significantly associated with T stage and TNM stage (*p* < 0.01). For the other clinical pathological characteristics, no significant association was found. However, Kaplan–Meier survival analysis showed that patients with high GPRIN1 expression had a worse prognosis (*p* < 0.001, Figure 1C).

**Table 1.** The relationship between GPRIN1 expression and clinical pathological characteristics in patients with GBC.

	GPRIN1 expression			
Parameters	*N*	Low	High	*p*-value
Sex				0.640
Male	17	7	10	
Female	46	16	30	
Age				0.868
≤65	31	11	20	
>65	32	12	20	
Tumor diameter				0.177
≤3 cm	15	7	8	
>3 cm	50	18	32	
T stage				0.007
T2	27	15	12	
T3–T4	36	8	28	
N stage				0.270
N0	44	18	26	
N1–N2	19	5	14	
M stage				0.424
M0	58	22	36	
M1	5	1	4	
TNM stage				0.017
Ⅰ–Ⅱ	26	14	12	
Ⅲ–Ⅳ	37	9	28	

**Figure 1.**
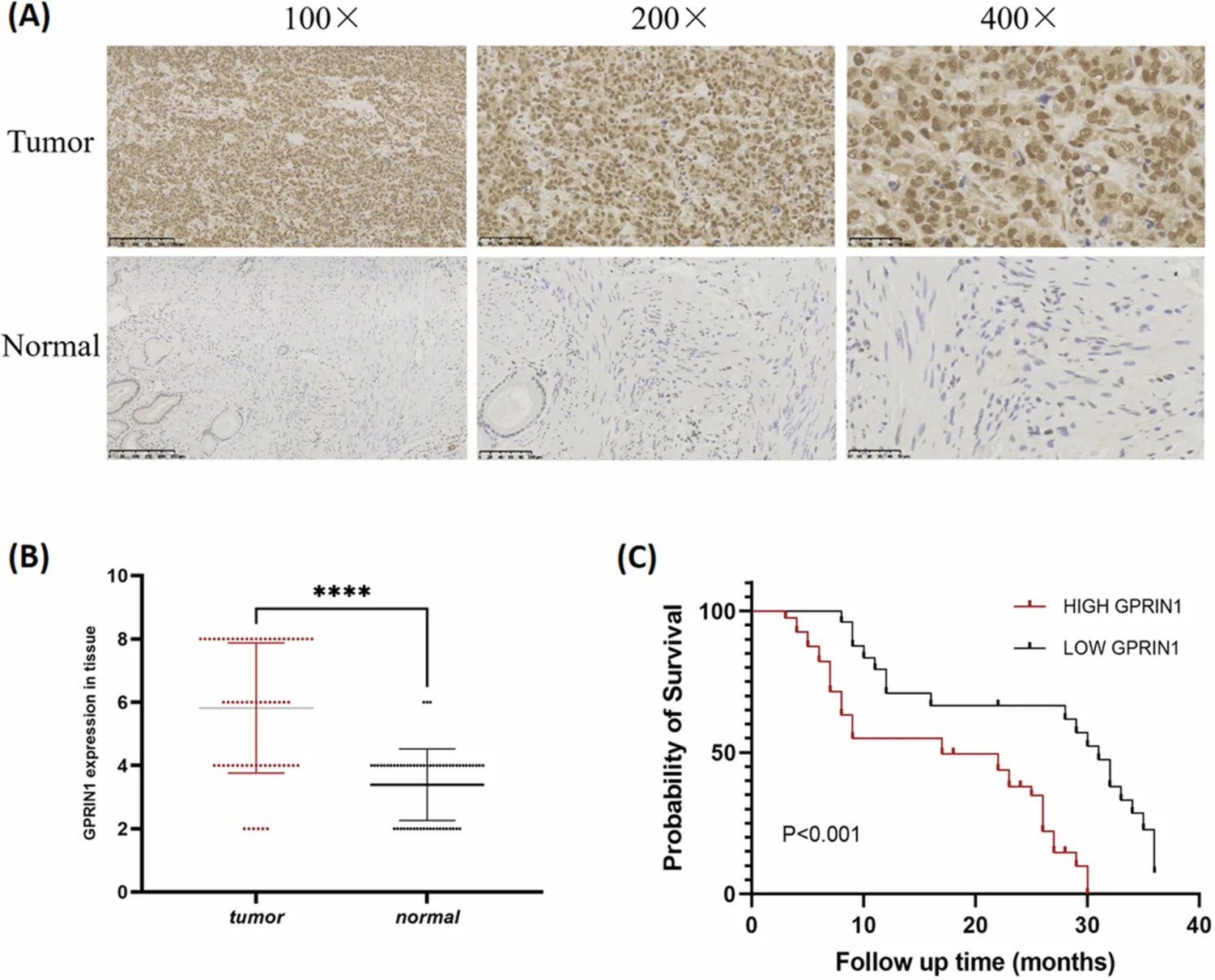
Increased GPRIN1 expression was associated with worsened prognosis in GBC patients. (A) GBC tissue with high GPRIN1 expression and normal tissue with low GPRIN1 expression. (B) Scatterplot showing the quantification of GPRIN1 protein levels in GBC tumor and normal tissues. (C) Kaplan–Meier analyses of overall survival to assess the prognostic value of GPRIN1 in GBC patients.

### GPRIN1 promotes GBC cell proliferation in vitro

Cell proliferation and MTT assays revealed that the proliferation of GBC cells was significantly inhibited after suppression of GPRIN1 expression (Figure 2A–D). After shRNA lentivirus infection of GBC-SD cells, the number of cells in the S phase was obviously decreased (*p* < 0.05), the number of cells in the G1 phase did not change (*p* > 0.05), and the number of cells in the G2/M phase was significantly increased (*p* < 0.05), suggesting that GPRIN1 was significantly related to the cycle of GBC-SD cells (Figure 2E). In addition, the number of apoptotic GBC-SD and NOZ cells increased significantly after shRNA lentivirus infection (*p* < 0.05), suggesting that GPRIN1 was significantly related to the apoptosis of GBC-SD and NOZ cells (Figure 2F and G; Supplementary Figure 1). Together, these findings suggested that silencing GPRIN1 expression inhibited GBC cell proliferation in vitro.

**Figure 2.**
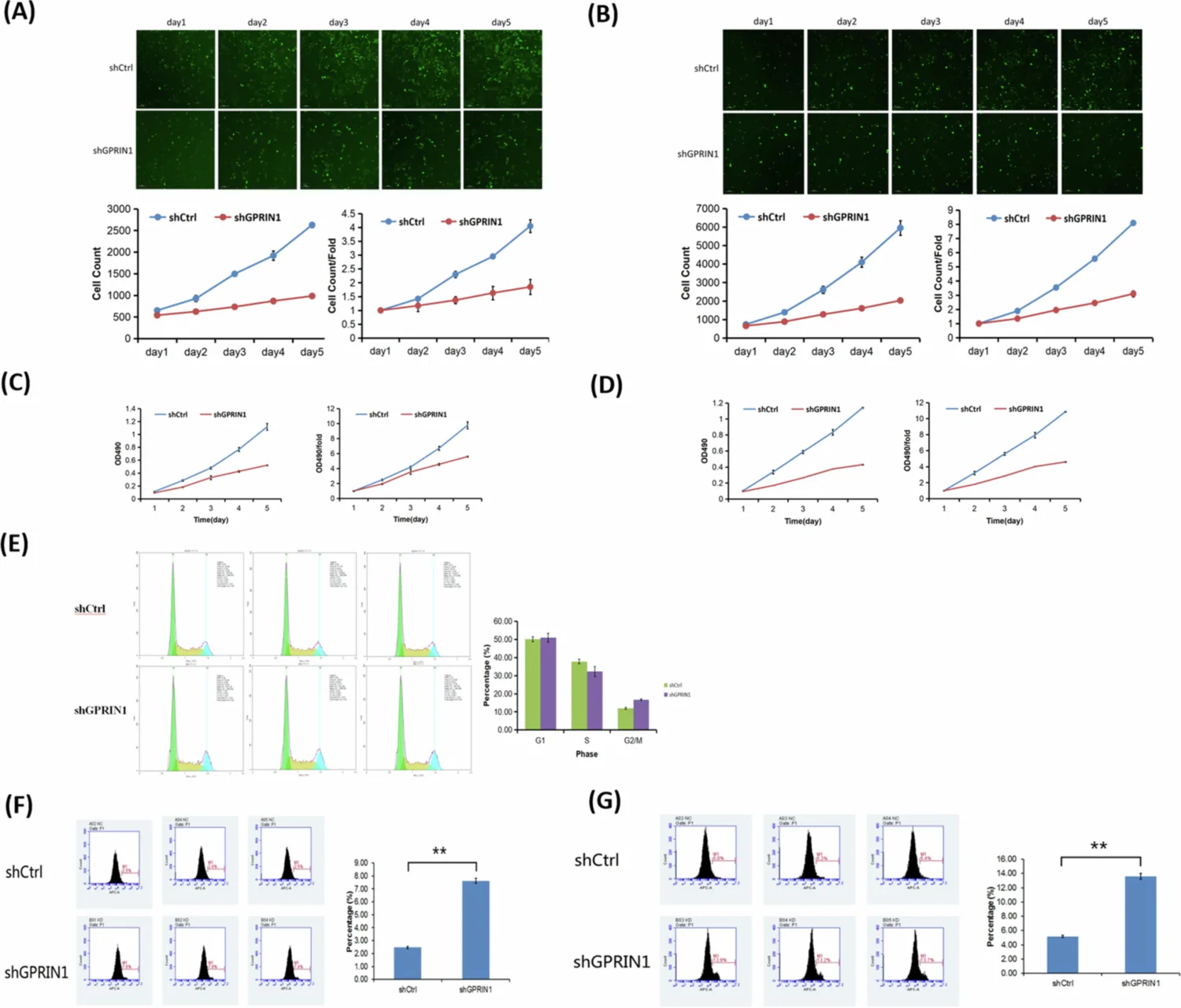
GPRIN1 promotes GBC cell proliferation in vitro. Celigo was detected continuously for 5 d, and the proliferation rate of GBC-SD (A) and NOZ cells (B) transfected with shRNA was significantly inhibited. An MTT assay indicated that the proliferative capacities of GBC-SD (C) and NOZ cells (D) transfected with shGPRIN1 were significantly inhibited. GPRIN1 knockdown via shRNA lentivirus alters cell cycle distribution in GBC-SD cells: S-phase cells decrease (*p* < 0.05), G2/M-phase cells increase (*p* < 0.05), with no significant change in G1-phase cells (*p* < 0.05) (E). The number of GBC-SD (F) and NOZ cells (G) undergoing apoptosis after transfection with shGPRIN1 was measured by flow cytometry.

### GPRIN1 promotes the migration and invasion of GBC cells in vitro

Cell invasion and migration assays were performed by transferring GBC cells to Transwell chambers coated with or without an extracellular matrix. Transwell assays coated without extracellular matrix showed that the migration capacities of GBC-SD and NOZ cells in the experimental group were significantly decreased upon GPRIN1 knockdown (*p* < 0.05 and *p* < 0.05) (Figure 3). Transwell assays coated with extracellular matrix showed that the invasive capacities of GBC-SD and NOZ cells in the experimental group were significantly decreased upon GPRIN1 knockdown (*p* < 0.05 and *p* < 0.05) (Figure 4). Together, these findings suggested that silencing GPRIN1 expression inhibited the migration and invasion of GBC cells in vitro.

**Figure 3.**
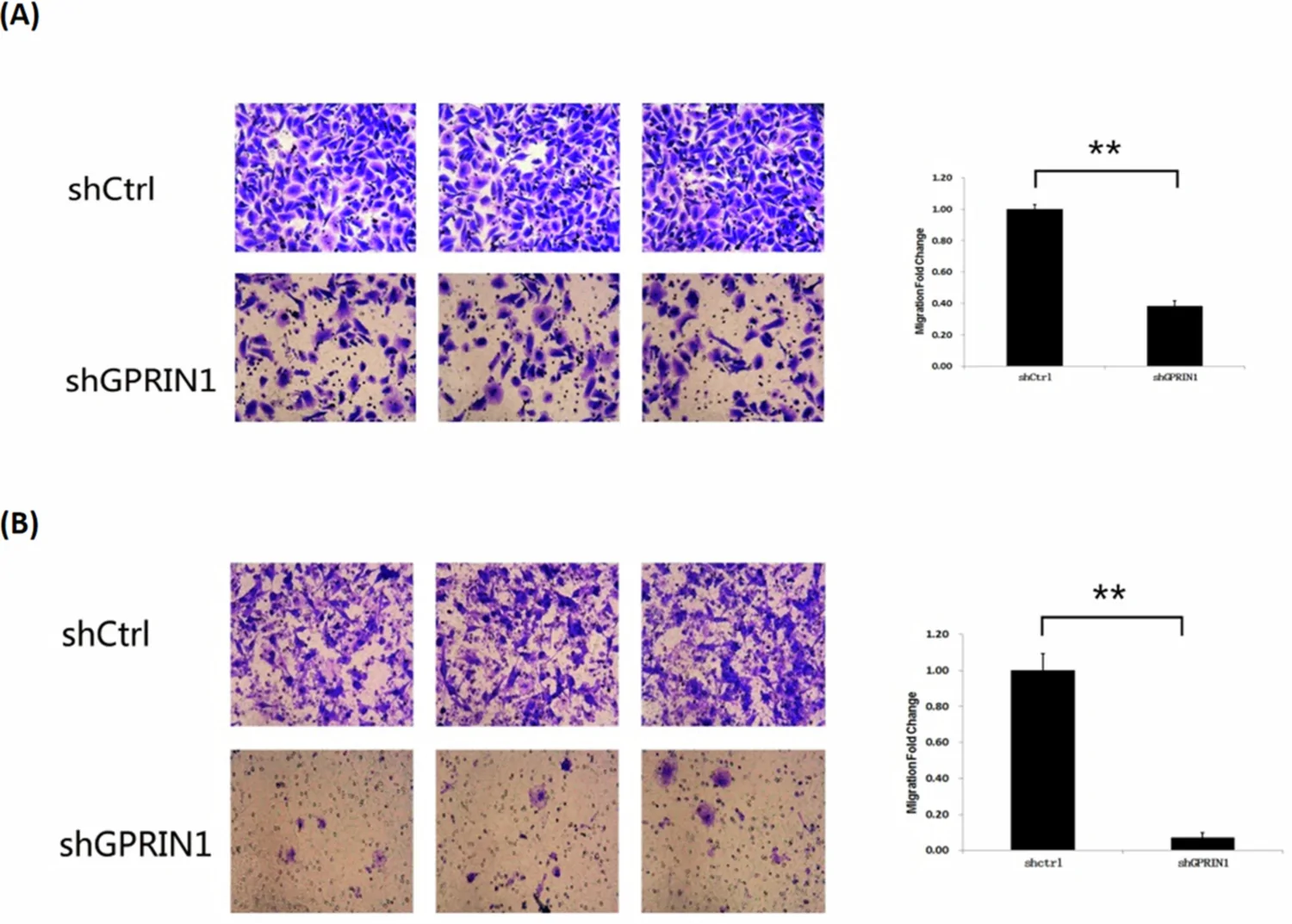
GPRIN1 promotes GBC cell migration in vitro. Transwell assays coated without extracellular matrix showed that the migratory capacities of GBC-SD (A) and NOZ (B) cells transfected with shGPRIN1 were significantly decreased (*p* < 0.05 and *p* < 0.05).

**Figure 4.**
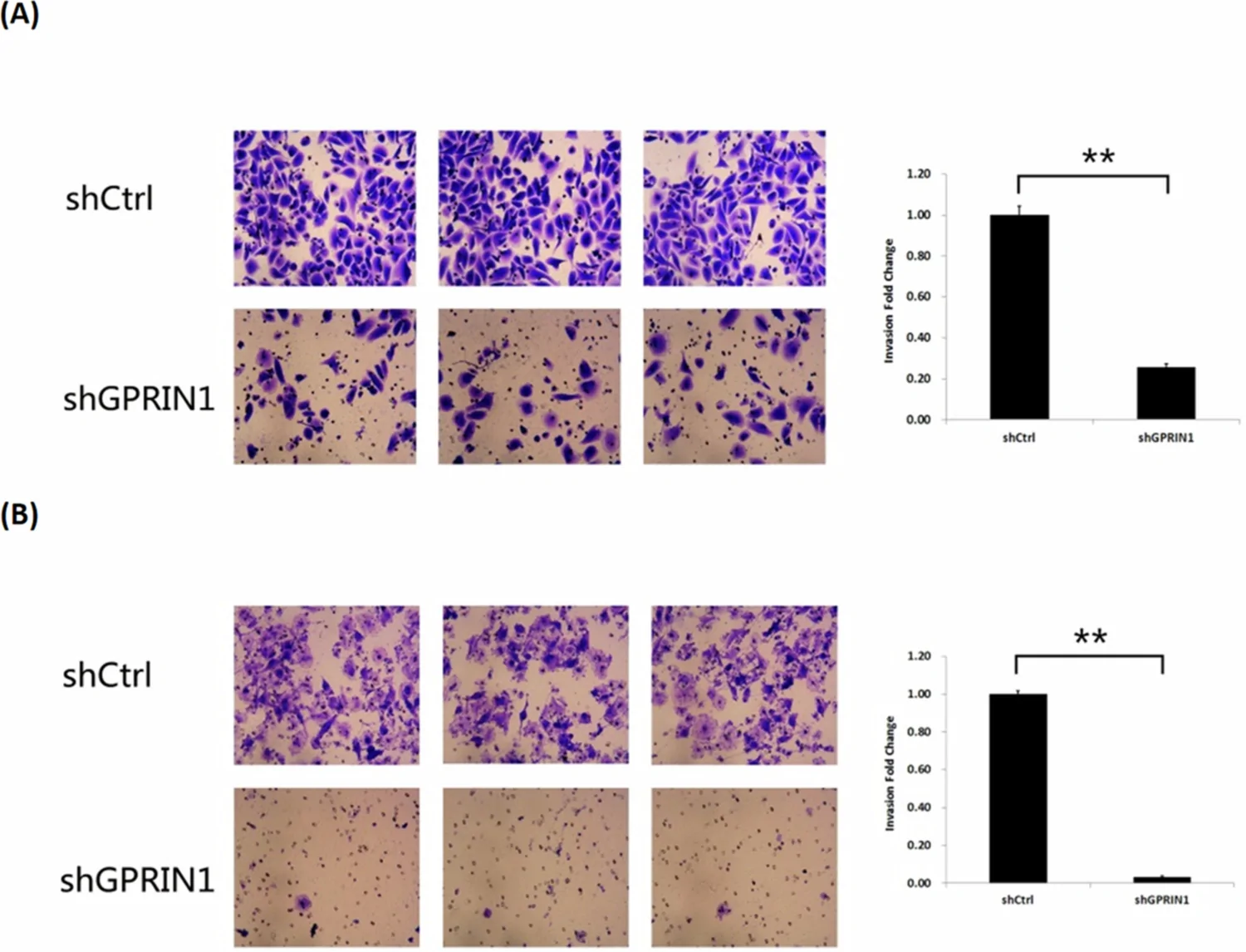
GPRIN1 promotes GBC cell invasion in vitro. Transwell assays coated with extracellular matrix showed that the invasive capacities of GBC-SD (A) and NOZ cells (B) transfected with shGPRIN1 were significantly decreased (*p* < 0.05, *p* < 0.05).

### GPRIN1 promotes cell proliferation in GBC in vivo

To further identify whether abnormal expression of GPRIN1 promoted tumorigenicity of GBC in vivo, a subcutaneous xenograft tumor model was established in nude mice. Two weeks after subcutaneous injection of the cells, subcutaneous tumors in the sh-GPRIN1 group were markedly smaller than those in the control group. Four weeks later, the difference was even more significant (Figure 5). Ultimately, the average volume and weight of the subcutaneous tumors formed by NOZ cells in the sh-GPRIN1 group were significantly smaller than those in the control group (*p* < 0.01) (Figure 6). Collectively, these results confirmed that high GPRIN1 expression reinforced the proliferative and invasive capacities of tumor cells in vivo, consistent with our previous findings in vitro.

**Figure 5.**
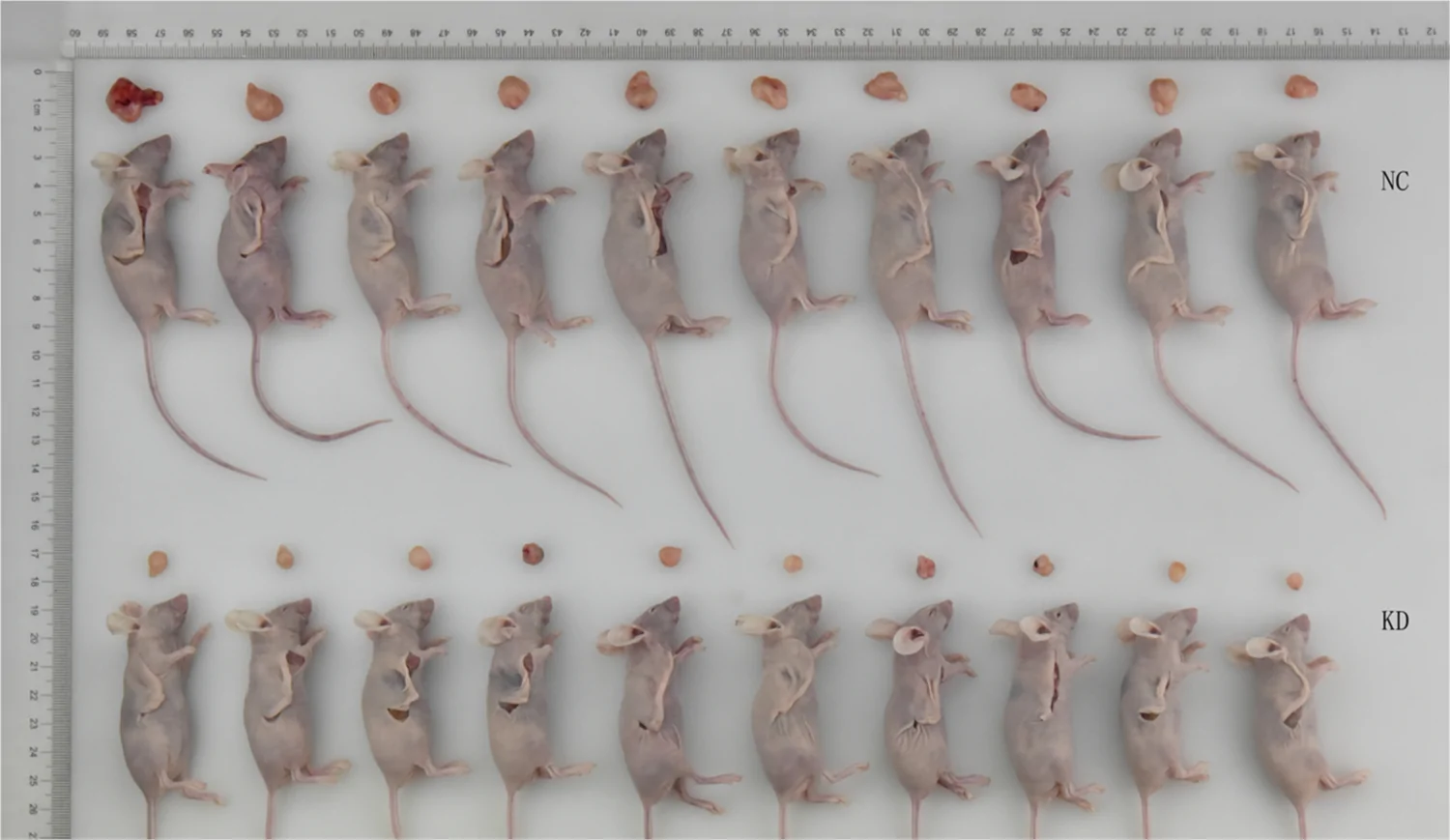
GPRIN1 promotes proliferation in GBC in vivo. A subcutaneous xenograft tumor model was established via subcutaneous injection of NOZ cells transfected with sh-GPRIN1 and empty vector constructs. Two weeks later, the subcutaneous tumors in the sh-GPRIN1 group were significantly smaller than those in the control group, and the difference was even more significant after 4 weeks.

**Figure 6.**
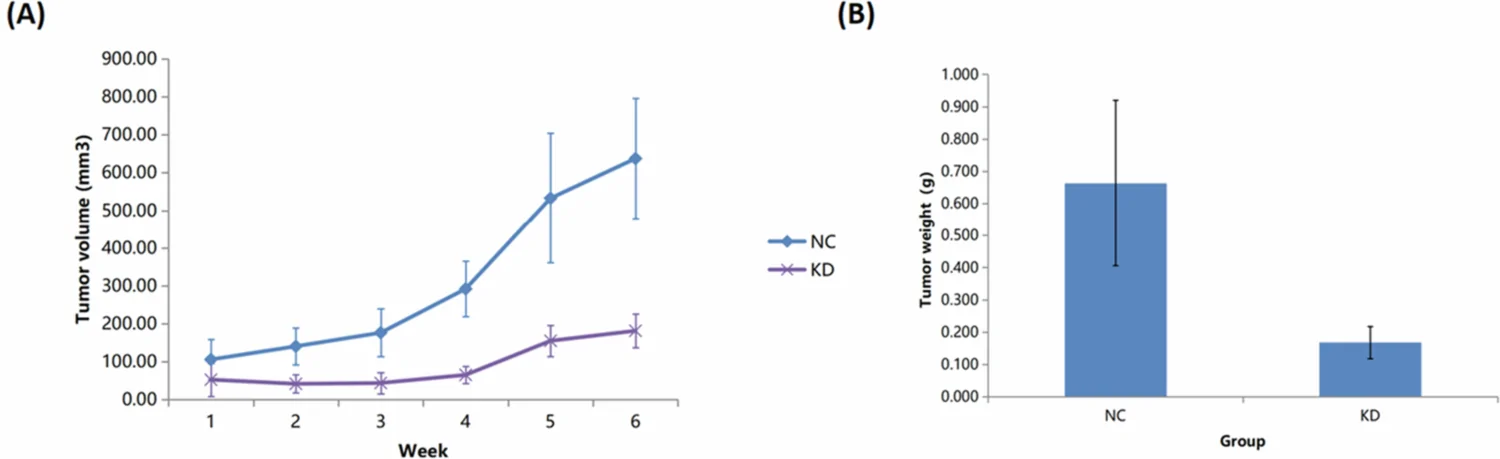
GPRIN1 promotes proliferation in GBC in vivo. The average volume (A) and weight (B) of the subcutaneous tumors formed by NOZ cells in the sh-GPRIN1 group were significantly smaller than those in the control group (*p* < 0.01).

### Co-IP assays

To further investigate the possible mechanism by which GPRIN1 facilitates GBC cell metastasis, co-IP assays and MS were performed to identify GPRIN1-interacting proteins. After immunoprecipitation, the Flag-tag was detected in the overexpression group (Figure 7A). The abovementioned lentivirus was used to infect NOZ cells. The aforementioned cell proteins were collected, and FLAG beads were used for co-IP. Flag-tagged bands were visualized by CBB staining in the overexpression group (Figure 7B). After liquid chromatography with tandem MS (LC–MS/MS) test, twenty-one proteins were detected in the control group, and 127 proteins were detected in the group overexpressing the 3× FLAG-GPRIN1 fusion gene. A bioinformatics analysis was performed with the proteins identified in the overexpression group, and the genes that might interact with GPRIN1 were predicted (Figure 7C). The genes that may interact with GPRIN1 were verified by Western blot analysis, which showed a direct interaction between GPRIN1 and CDK1 (Figure 7D). It was speculated that GPRIN1 induces changes in the cell functional phenotype by regulating the expression of the downstream CDK1 protein.

**Figure 7.**
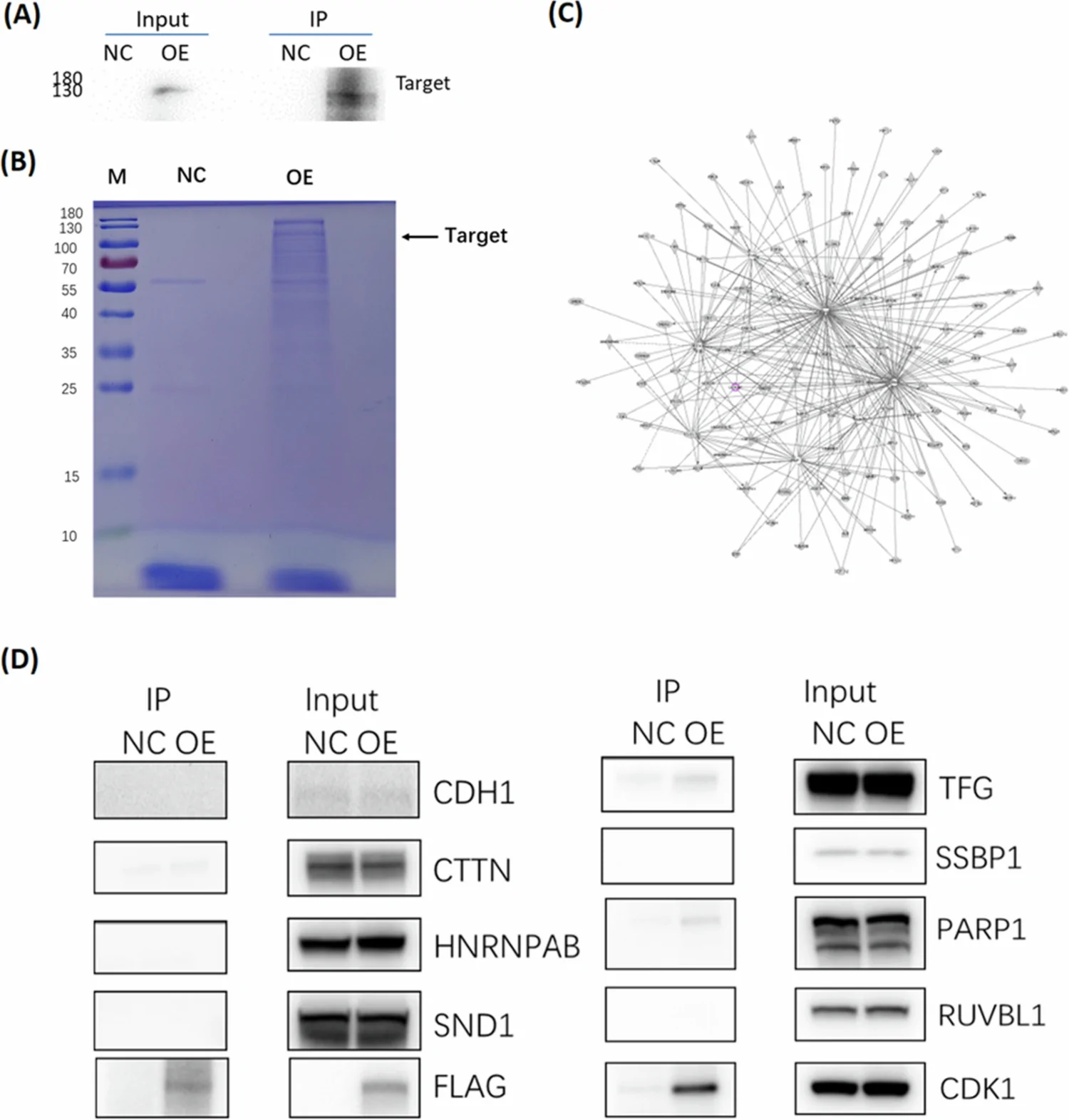
Coimmunoprecipitation (co-IP) assay. (A) In the whole-cell lysate (input) protein samples, the Flag tag was successfully detected in the overexpression (OE) group; after IP, the Flag tag was detected in the OE group. (B) After the protein complex was purified, SDS–PAGE and Coomassie Brilliant Blue staining were performed. (C) A bioinformatics analysis was performed with proteins identified in the OE group, and the gene products that might interact with GPRIN1 were predicted. (D) Western blots showing a direct interaction between GPRIN1 and CDK1.

### Effect of CDK1 functional recovery on GPRIN1

Cell proliferation, MTT and Transwell assays were performed to verify the functional recovery effect of CDK1 on GPRIN1. A cell proliferation assay revealed that the proliferation rate of the cells in the knockdown (KD)+negative control (NC) group was significantly lower than that of the NC+NC group (*p* < 0.05); the cell proliferation trend of the KD+overexpression (OE) group led to significant recovery compared with that in the KD+NC group (*p* < 0.05) (Figure 8A). MTT assays also resulted in similar conclusions (Figure 8B). Transwell assays showed that the migration capacities of the cells in the KD+NC group were lower than those in the NC+NC group (*p* < 0.05); the migration capacities of the cells in the KD+OE group were higher than those in the NC+NC group (*p* < 0.05) (Figure 8C). Together, these findings suggested that CDK1 exerted a functional recovery effect on GPRIN1.

**Figure 8.**
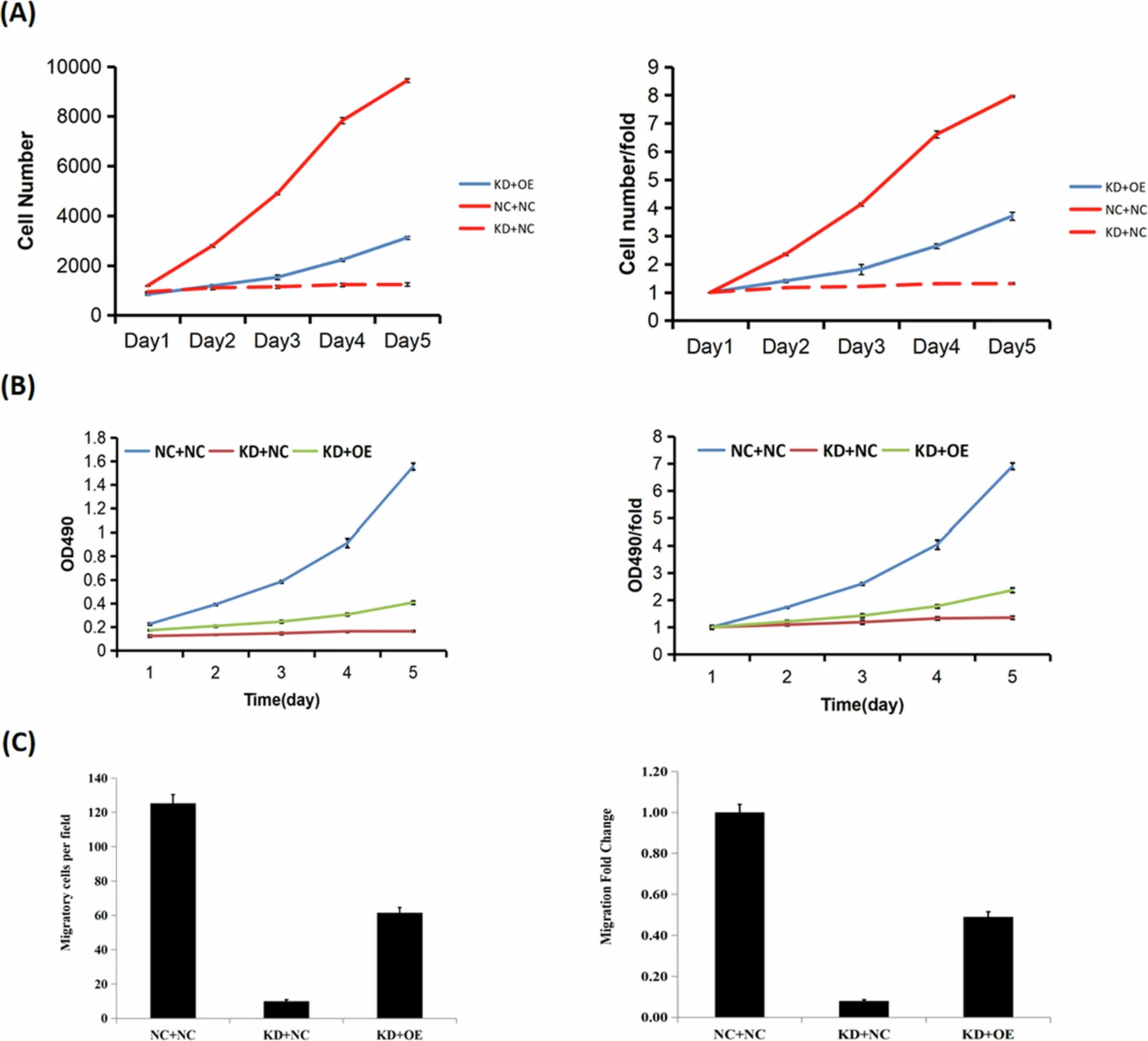
Effect of CDK1 functional recovery on GPRIN1. (A) A cell proliferation assay revealed that the proliferation rate of the cells in the KD+NC group was significantly lower than that of the NC+NC group (*p* < 0.05); the cell proliferation trend of the KD+OE group showed significant recovery compared with that of the KD+NC group (*p* < 0.05). (B) MTT assays also resulted in similar conclusions. (C) Transwell assays showed that the migratory capacities of the cells in the KD+NC group were lower than those in the NC+NC group (*p* < 0.05); the migratory capacities of the cells in the KD+OE group were higher than those in the NC+NC group (*p* < 0.05). KD: knockdown, NC: Negative control, OE: Overexpress.

### The identification of differentially expressed proteins and phosphorylated peptides

We compared the protein and phosphorylated peptide expression differences between the two groups. A total of 439 proteins were differentially expressed, including 198 proteins with upregulated expression and 241 proteins with downregulated expression. Moreover, a total of 1823 phosphorylated peptides were differentially expressed, including 1228 phosphorylated peptides with upregulated expression and 595 phosphorylated peptides with downregulated expression. A volcano plot visually showed the proteins and phosphorylated peptides differentially expressed between the two samples (Figure 9A and B). Heatmaps were generated to show the differential expression of these proteins (Figure 9C) or phosphorylated peptides (Figure 9D).

**Figure 9.**
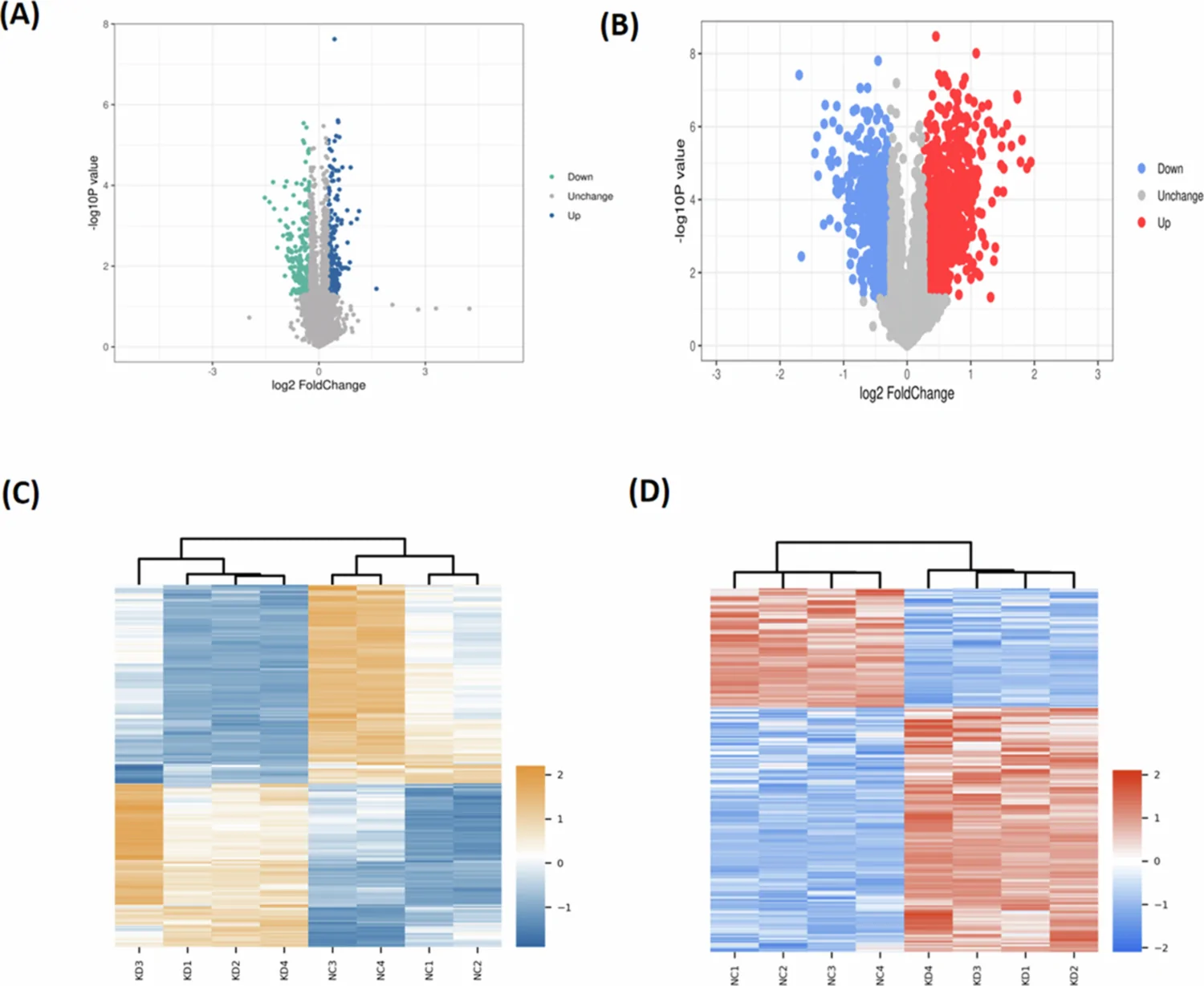
Identification of differentially expressed proteins and phosphorylated peptides. Volcano plot showing differentially expressed proteins (A) and phosphorylated peptides (B). Heatmap showing the quantitative proteome comprising differentially expressed proteins (C) and phosphorylated peptides (D).

We performed a proteomic analysis of differentially expressed proteins and phosphorylated peptides separately, and a combined analysis was subsequently performed in the following section.

### GO functional enrichment analysis of proteins with differentially expressed phosphorylated peptides

In the biological process (BP) analysis, we found that the two groups of proteins with differentially expressed phosphorylated peptides were mainly involved in cellular processes and biological regulation. In the molecular function (MF) analysis, both groups of proteins mainly showed binding and catalytic activity. In the cellular component (CC) analysis, the functions of the two groups were basically the same (Figure 10A).

**Figure 10.**
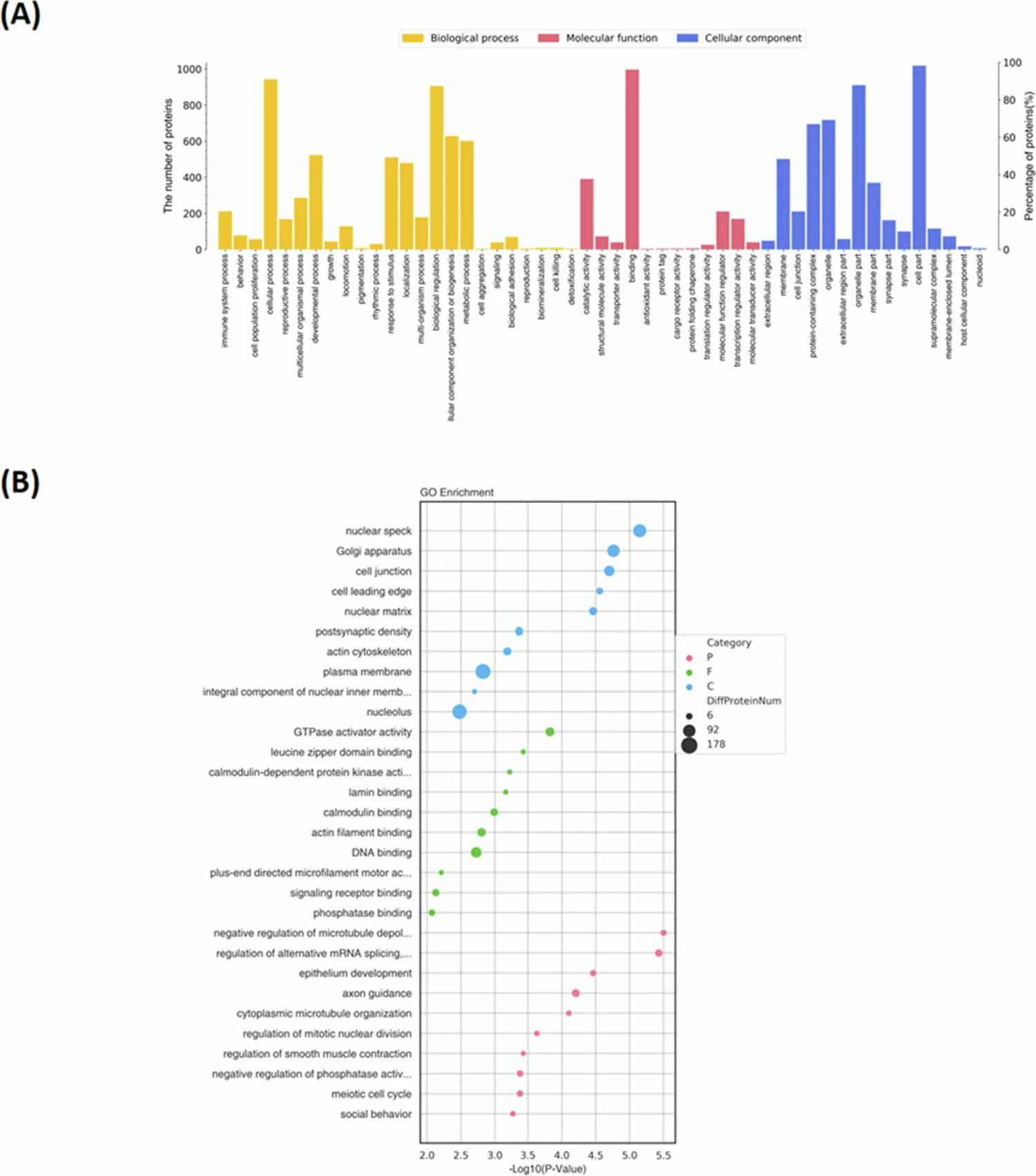
GO functional enrichment analysis. (A) GO annotation results. (B) GO functional enrichment analysis: Bubble color indicates the GO term name (P: biological process, F: molecular function, C: cellular component); bubble size indicates the number of proteins with differentially phosphorylated peptides enriched in the GO function.

GO functional enrichment analyses indicated that the expressed proteins with differentially expressed phosphorylated peptides were mainly enriched in negative regulation of microtubule depolymerization and regulation of alternative mRNA splicing via the spliceosome. In the MF category, the expressed proteins were enriched in GTPase activator activity and leucine zipper domain binding. Additionally, CC analysis revealed that the expressed proteins were significantly enriched in the nuclear speck and Golgi apparatus. (Figure 10B).

### KEGG enrichment, domain enrichment and subcellular structure localization of proteins with differentially expressed phosphorylated peptides

The KEGG pathway enrichment analysis revealed that these proteins were mainly implicated in phototransduction-fly, viral myocarditis and adherens junction. The 10 pathways with the highest enrichment scores are shown in Figure 11. A protein domain enrichment analysis led to the identification of 10 significantly enriched domains (Figure 12), mainly including the PH-like domain superfamily and pleckstrin homology domain. A subcellular localization analysis was shown in Figure 13, the differentially expressed proteins in the two comparison groups were mainly localized in the nucleus (69.8%) and cytosol (15.6%).

**Figure 11.**
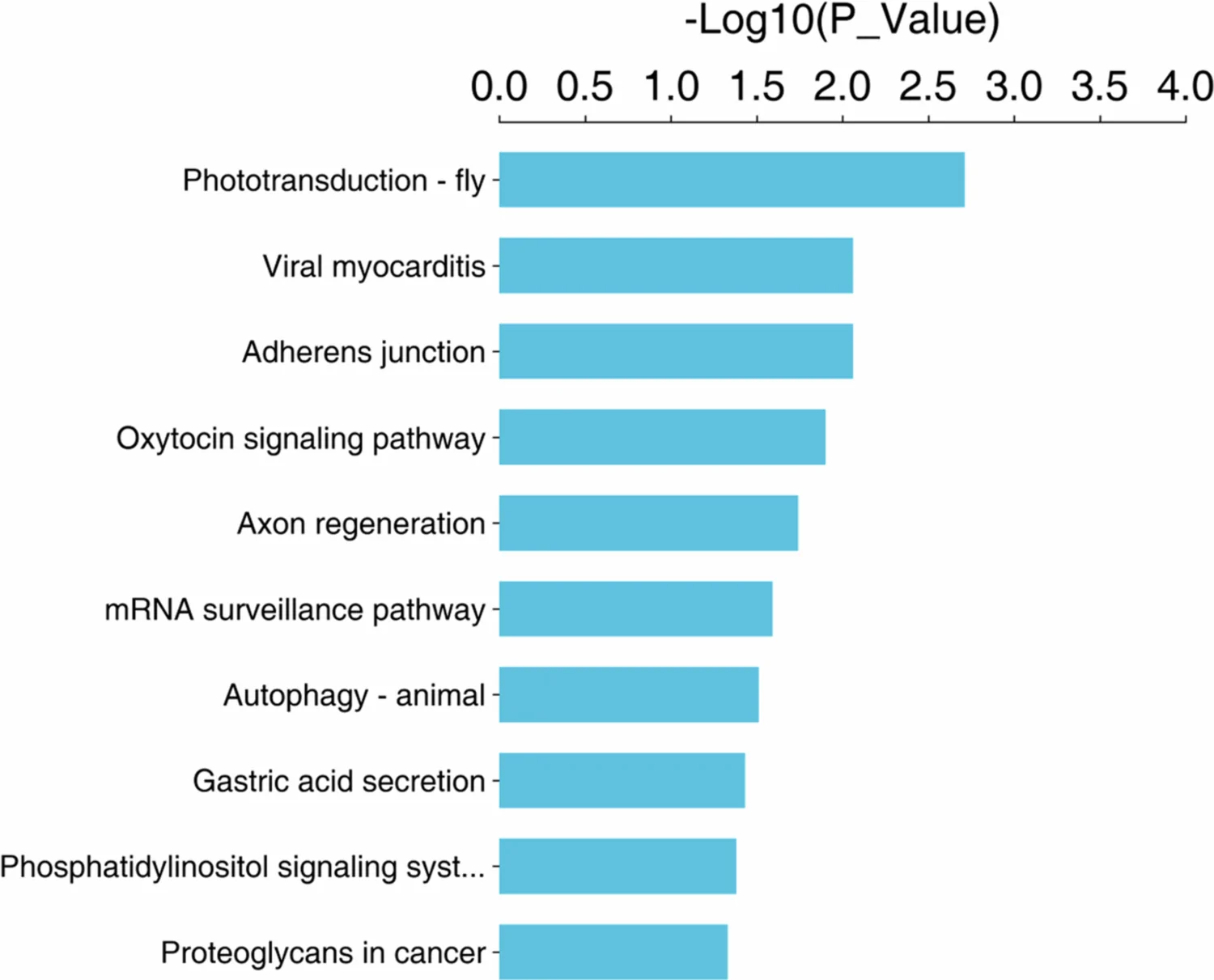
KEGG pathway enrichment analysis. Statistical histogram showing the significantly enriched KEGG pathways consisting of proteins with differentially expressed phosphorylated peptides (KD vs. NC).

**Figure 12.**
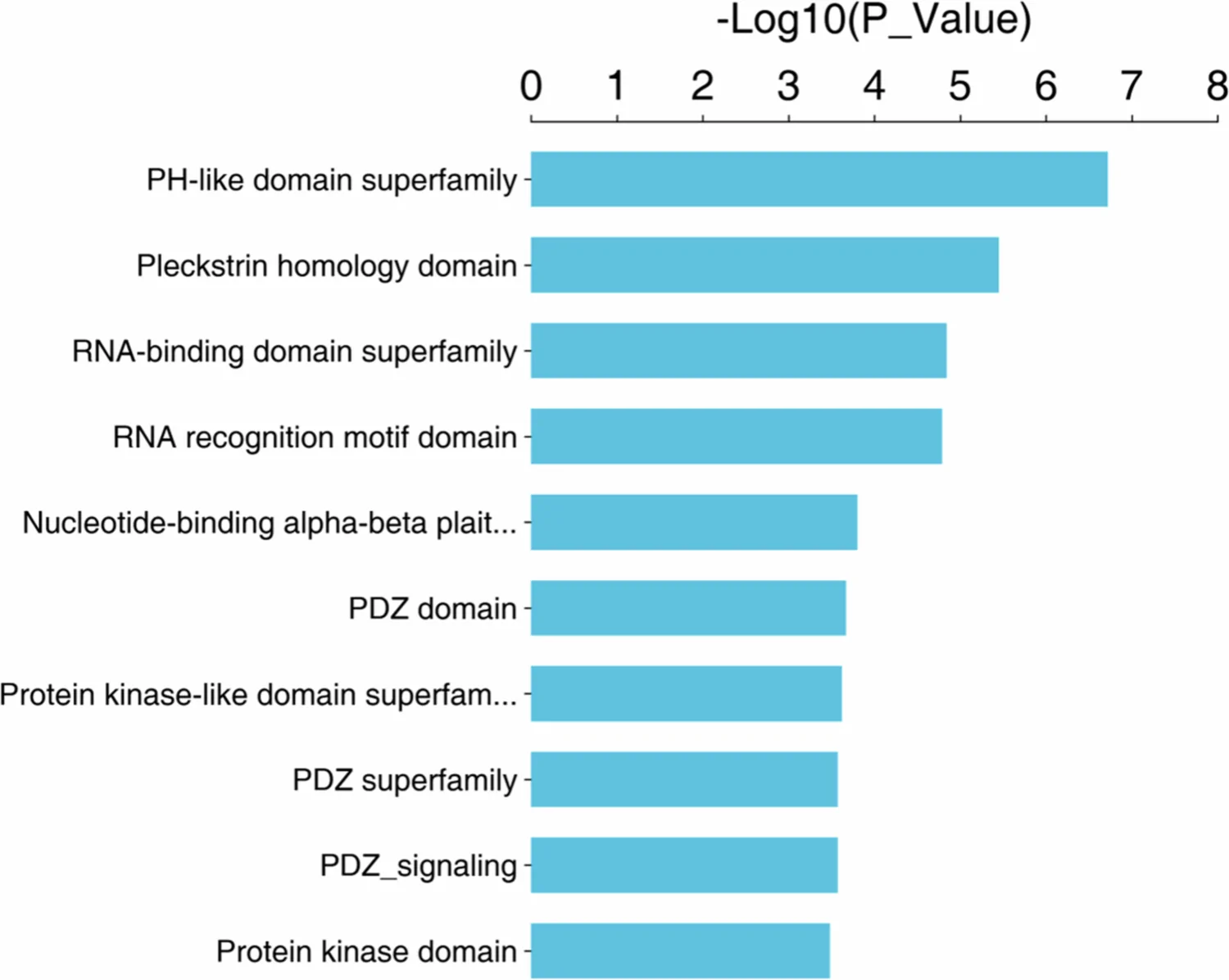
Domain enrichment analysis. Statistical histogram showing significantly enriched protein domains in differentially expressed phosphorylated peptides (KD vs. NC).

**Figure 13.**
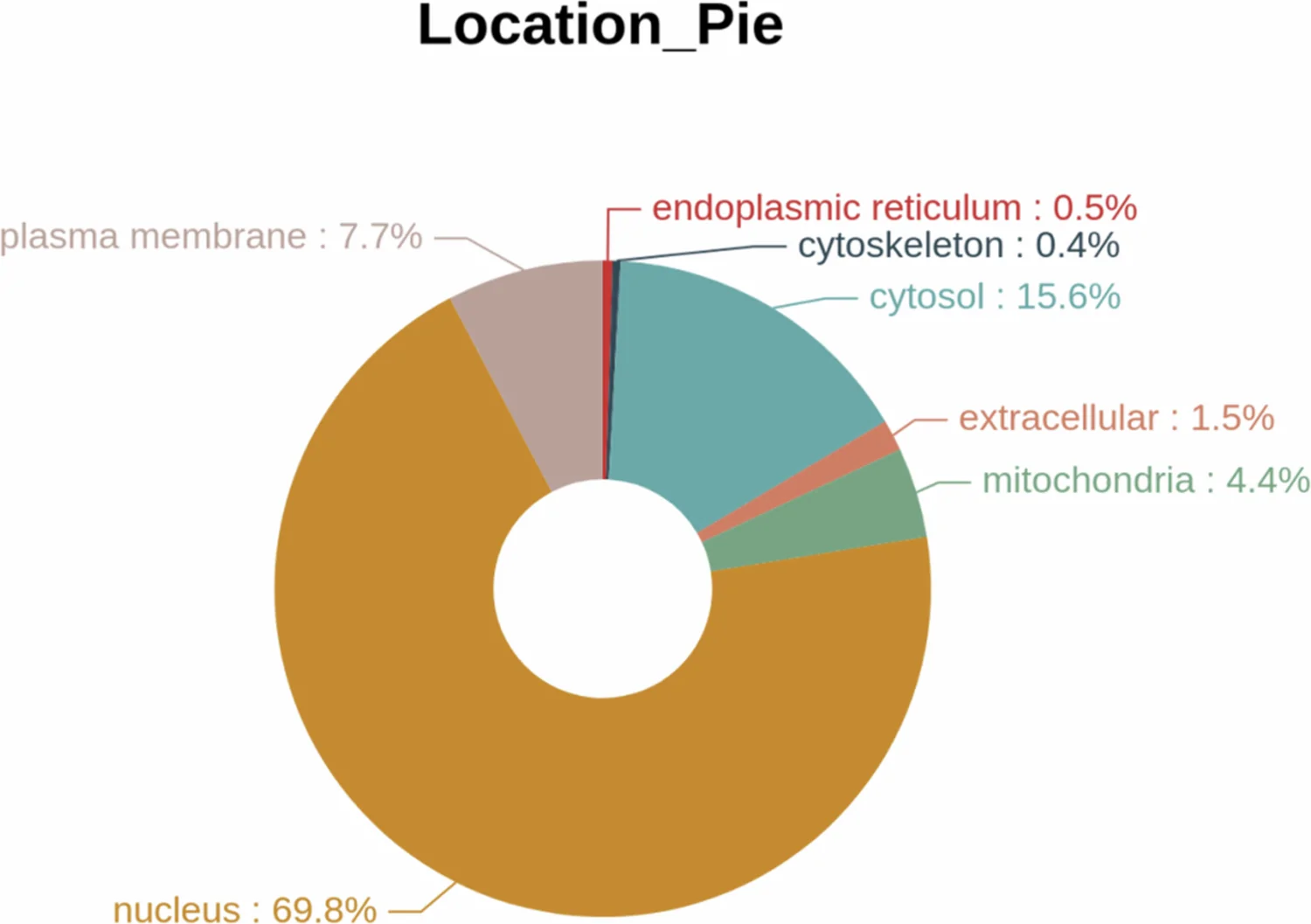
Subcellular structure localization analysis. Statistical pie chart showing the subcellular localization of proteins with differentially expressed phosphorylated peptides (KD vs. NC).

### Upstream kinase-substrate interaction analysis based on phosphorylation sites

To better understand the process of signaling regulation and signal transduction, the upstream kinases of each phosphorylation site were predicted and analyzed. The network analysis of the interaction between phosphorylated upstream kinases and their substrates are shown in Figure 14. CDK1 interacts with CDK2, CDK7 and EEF2K kinases. KIF11_T926 was in an activated state after being phosphorylated, and RBI_S249, PIK3C2A_S259, LBR_S71, NPMI_T199, MAP4_S696, and MAP4_S787 were in an inhibitory state after being phosphorylated.

**Figure 14.**
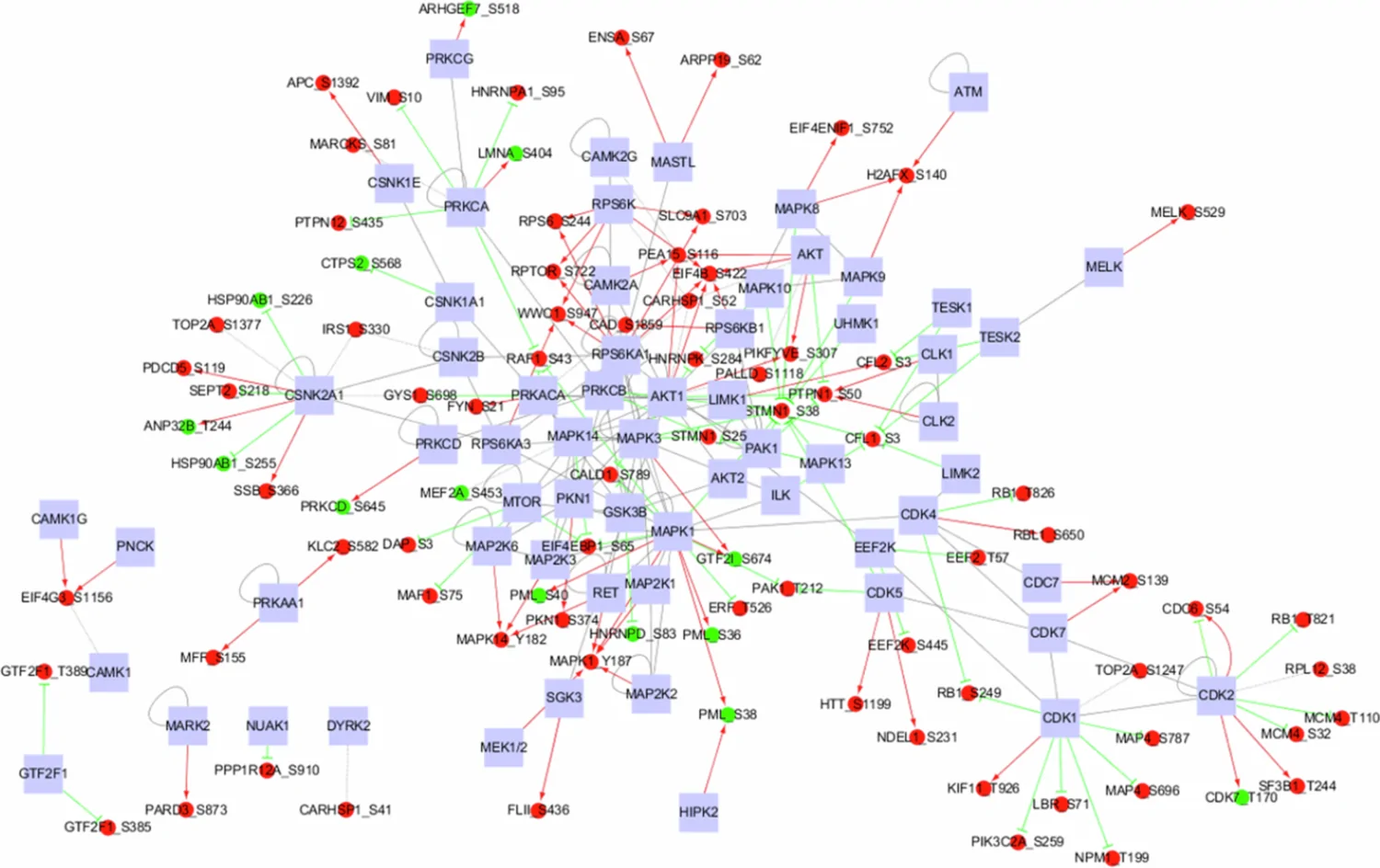
Network analysis. The purple squares represent upstream kinases, the red circles represent downstream substrates with expression that is upregulated after phosphorylation, the green circles represent downstream substrates with expression that is downregulated after phosphorylation, and the solid red lines represent proteins in the activated state after phosphorylation. The green solid line represents the inhibitory state after a substrate is phosphorylated; the gray dashed line represents an unclear state after the substrate is phosphorylated; and the gray solid line represents the interaction between upstream kinases.

## Discussion

The GPRIN protein family members, consisting of three gene products (GPRIN1, GPRIN2, and GPRIN3), are highly expressed in the brain and bind selectively to activated forms of Gαo, Gαz, and Gαi, which can mediate signal transduction through G-protein-coupled receptors and sequential intracellular targets.^12^ ^,^ ^13^ GPRIN1 is a predominantly membrane-bound protein highly enriched in neurite growth cones and neurite outgrowths1.^5^ ^,^ ^12^ Studies have shown that GPRIN1 not only plays a crucial role in neuronal network development but is also involved in tumor proliferation, invasion, and metastasis.^7^ Some studies showed that GPRIN1 was associated with poor prognosis and tumor immune cell invasion in kidney renal papillary cell carcinoma and lung adenocarcinoma, and the expression of GPRIN1 was significantly upregulated in various types of human tumors.^9^ However, another study showed that GPRIN1 inhibited gastric carcinoma cell proliferation, migration, and invasion.^14^ Nevertheless, the function of GPRIN1 in GBC is still unclear.

In this study, we found that GPRIN1 was highly expressed in GBC, and increased GPRIN1 expression was associated with poor prognosis. To further understand the biological function of GPRIN1 in GBC, shRNA was used to knockdown GPRIN1 expression in GBC-SD and NOZ cells. We found that GPRIN1 promoted the proliferation, migration and invasion of GBC in vitro. To determine whether overexpression of GPRIN1 promotes GBC tumorigenicity in vivo, a subcutaneous xenograft tumor model was established in nude mice. The in vivo experiments confirmed that high GPRIN1 expression reinforced the proliferative and invasive capacities of GBC cells, consistent with our previous findings obtained in vitro. To better investigate the possible mechanism by which GPRIN1 facilitates GBC metastasis, co-IP assays and MS were performed to identify GPRIN1-interacting proteins. A bioinformatics analysis was performed with the proteins identified in the overexpression group, and the genes that might interact with GPRIN1 were predicted. The genes that may interact with GPRIN1 were verified by Western blot analysis, which showed that a direct interaction between GPRIN1 and CDK1.

CDKs are critical for controlling all aspects of cell division and modulating transcription in response to several extracellular and intracellular cues.^10^ ^,^ ^15^ CDK1, with a molecular weight of 34 kDa, is encoded by cell division cycle gene 2 and is a member of the Ser/Thr protein kinase family.^11^ As the first CDK to be identified, CDK1 is conserved in all organisms, playing important roles in mitosis and driving cells into the S phase in the absence of CDK2.^10^ In recent years, studies have shown that CDK1 is overexpressed and is valuable for estimating the prognosis of different kinds of cancers, such as ovarian cancer, breast cancer, hepatocellular cancer, gastric cancer and lung cancer.^11^

Our proteome analysis revealed the involvement of CDK1 in many biological processes and cellular pathways. Our analysis showed that CDK1-associated phosphorylation plays a potential role in the negative regulation of microtubule depolymerization and the regulation of alternative mRNA splicing via the spliceosome. In addition, our results revealed previously unknown potential roles played by CDK1 in adherens junctions, the phosphatidylinositol signaling system, and proteoglycans in cancer, suggesting a broad regulatory role played by CDK1 in various cellular functions. Adherens junctions are vital for maintaining epithelial homeostasis and integrity, and their dysfunction is reportedly linked to tumor progression.^16^ The phosphatidylinositol signaling system is significantly involved in the development and progression of several cancers by altering the signaling of major oncogenic pathways implicated in cancer, such as the PI3K/Akt/mTOR, RAS/RAF/MAPK/ERK, and JAK/STAT pathways.^17^ As major constituents in the extracellular matrix, proteoglycans play important roles in cancer angiogenesis, proliferation, invasion, and metastasis through interactions with a variety of cytokines.^18^ ^,^ ^19^ Additionally, based on proteomic data, we hypothesize that CDK1 interacts with CDK2, CDK7, and EEF2K kinases. These potential associations may offer unique perspectives and point to resources for future investigation into GBC-related signaling pathways. However, further experimental validation is necessary to establish the functional relevance of these interactions in GBC progression.

Several limitations should be acknowledged in this study. Although our findings suggest a functional interaction between GPRIN1 and CDK1 in promoting gallbladder cancer progression, we must emphasize that the causal mechanism remains incompletely defined, and CDK1-inhibition functional experiments have not been performed in the present work. While co-immunoprecipitation and proteomics data support their association, definitive confirmation of CDK1 as an obligate mediator of GPRIN1-driven oncogenesis will require: (1) CDK1 knockdown/rescue experiments combined with phenotypic assessments (e.g., proliferation, migration), and (2) pharmacological inhibition (e.g., RO-3306) to rigorously test the necessity of CDK1 activation. Additionally, while our proteomics/phosphoproteomics analyses identified promising candidates and pathways, these results primarily serve as a hypothesis-generating foundation. Future work will prioritize orthogonal validation approaches (e.g., qRT-PCR, Western blot) for top-ranked targets, particularly those within CDK1-related signaling networks, to establish their biological relevance in GBC pathogenesis.

## Conclusion

In summary, this study demonstrates that elevated GPRIN1 expression correlates with poor prognosis in GBC patients and promotes tumor cell proliferation, migration, and invasion in vitro and in vivo. While our integrated proteomics and co-immunoprecipitation data suggest a functional interaction between GPRIN1 and CDK1, the mechanistic relationship remains incompletely defined; current evidence supports that GPRIN1 may influence CDK1-related pathways rather than directly regulating CDK1 expression. Notably, phosphoproteomic analyses identified potential interactions between CDK1 and kinases such as CDK2, CDK7, and EEF2K, but these associations are explicitly framed as hypotheses requiring experimental validation to establish their biological significance in GBC pathogenesis. Although our findings highlight GPRIN1-CDK1 signaling as a potential therapeutic axis, clinical translation remains exploratory due to the absence of functional inhibition studies. Future work will prioritize mechanistic validation of these targets and preclinical evaluation of their therapeutic potential.

## Materials and methods

### Patients and tissue samples

In this study, GBC tissues were obtained from 65 patients who had undergone surgical resection at Zhongshan Hospital between 2015 and 2017. Of these, two cases with pT1 stage were excluded because of the diagnostic variability of pT1 cases; 61 patients underwent radical cholecystectomy including cholecystectomy, partial liver resection and extended lymphadenectomy; and 2 patients with distant metastasis underwent laparoscopic lymph node biopsy and palliative cholecystectomy. In addition, chronic cholecystitis specimens were obtained from 59 patients who had undergone surgical resection at Zhongshan Hospital in 2015. Selection criteria were: primary tumors arising from the gallbladder, a definite histological diagnosis, and good preservation of the samples. We retrospectively reviewed the clinicopathological and baseline demographic characteristics of the patients, including sex, age, tumor diameter, and tumor TNM stage, on the basis of their medical records. Histopathological examinations and TNM staging were confirmed by two experienced pathologists from the Pathology Department of Zhongshan Hospital who followed the AJCC 8th edition staging system. Follow-up data were available in all cases, ranging from 2 months to 60 months. Overall survival was determined from the date of surgery to death or the last follow-up visit. Written informed consent was obtained from each patient prior to surgery. The study was approved by the Clinical Research Ethics Committee of Zhongshan Hospital.

### Immunohistochemistry and staining evaluation

Tissue slides were deparaffinized with xylene and rehydrated in graded ethanol solutions. Antigen retrieval was achieved in 0.01 M citrate buffer (pH 6) for 20 min in a microwave oven. Slides were blocked with 10% fetal calf serum (FCS) in TBS for 30 min at room temperature and then incubated with the appropriate primary antibody overnight. After washing with TBS, sections were incubated with secondary antibody. Vulcan Fast Red Chromogen Kit 2 was used for color development. Finally, sections were developed with diaminobenzidine and counterstained with hematoxylin. All images were taken with an inverted optical microscope (XDS-100; Shanghai Caikon Optical Instrument Co., Ltd., Shanghai, China). The stained sections were evaluated and graded by two independent pathology experts. The expression score of GPRIN1 was calculated by multiplying the estimate of the staining intensity (staining intensity score) by the percentage of positive cells (quantity score). The staining intensity was scored as follows: 0 (no staining), 1 (weak staining), 2 (moderate staining), and 3 (strong staining). The percentage of GPRIN1-positive cells was scored as follows: 0 (0%), 1 (1%–25%), 2 (26%–50%), 3 (51%–75%), and 4 (76%–100%).

### Cell lines and culture conditions

The GBC-SD and NOZ human GBC cell lines and the 293T human embryonic kidney cell line were purchased from the Institute of Biochemistry and Cell Biology, Chinese Academy of Sciences, Shanghai, China. All the cells were cultured in RPMI 1640 media (Gibco; Thermo Fisher Scientific, Inc., USA) containing 10% FCS (Ausbian, Sydney, Australia). The cells were incubated in humidified air containing 5% CO_2_ at 37 °C.

### Lentiviral transfection

Generay (Shanghai, China) assisted in the design and production of GPRIN1 and CDK1 short hairpin RNA (shRNA) and the lentiviral vector GV610 containing GPRIN1. GBC cells were cultured in 6-well plates until 80% confluent. Medium containing green fluorescent protein-expressing lentivirus was collected and added to each well depending on the multiplicity of infection of the GBC cells. After 72 h, the expression of GFP was roughly monitored with a fluorescence microscope. The cell transfection efficiency was further examined by quantitative real-time PCR (qRT–PCR) and Western blotting (Supplementary Figure 2). The target sequence of GPRIN1 was 5′ GAGTCCTGTTACCA -CCACAAA 3′, and the control target sequence was 5′ TTCTCCGAACGTGTCACGT 3′. The target sequence of CDK1 was 5′ TACCTATG GAGTTGTGTA -TAA 3′, and the control target sequence was 5′ TTCTCCGAACGTGTCACGT 3′.

### Quantitative real-time PCR

Total RNA was extracted and purified using standard TRIzol RNA extraction according to TRIzol manufacturer's instructions, and reverse transcription was performed with M-MLV (Promega, Madison, WI, USA). Quantitative real-time PCR analysis was performed on an MX3000P Quantitative PCR system (Agilent Technologies, Santa Clara, CA, USA) with SYBR Green PCR Master Mix (Takara Biotec, Tokyo, Japan). The primers used for PCR were as follows: GPRIN1, forward: GTGGGAAATGTAGAAACTGTGC, and reverse: TCTGTCTTTGCTGAAGCCGT, 131 bp; GAPDH, forward: TGACTTCAACAGCGACACCCA, and reverse: CACCCTGTTGCTGTAGCCAAA, 121 bp. Cycling conditions were as follows: initial activation at 94 °C for 3 min; followed by 22 cycles of 94 °C for 30 s, 55 °C for 30 s, and 72 °C for 30 s. The relative expression levels of target mRNA in cells were analyzed using the 2^−^^ΔΔCt^ method.

### Co-IP assay

The gene sequence encoding the 3× FLAG tag was fused to the 5′ end of GPRIN1 by PCR technology to generate 3× FLAG-GPRIN1, which was inserted into a lentiviral expression vector to express plasmid p3× FLAG-GPRIN1. A lentiviral empty vector and p3× FLAG-GPRIN1 plasmid were cotransfected with helper plasmid into NOZ cells. The 3× FLAG-GPRIN1- interacting complex was purified using anti-FLAG magnetic beads (Sigma, A2220) according to the manufacturer's protocol.

### Western blot analysis

Proteins were extracted from the NOZ cells transfected with sh-GPRIN1 (negative control shRNA) using RIPA lysis buffer (Biyuntian, Jiangsu, China). A BCA protein assay kit (Biyuntian, Jiangsu, China) was utilized to quantify the protein. Proteins were separated by SDS–PAGE and then transferred onto a polyvinylidene difluoride membrane (Millipore, Billerica, MA, USA). The membrane was blocked with 5% nonfat milk in Tris-buffered saline Tween (TBST) and incubated at 4 °C overnight with primary antibodies. The following primary antibodies were used: anti-CDH1 (#3195s, CST), anti-CTTN (ab81208, Abcam), anti-HNRNPAB (ab199724, Abcam), anti-SND1 (ab65078, Abcam), anti-PARP1 (#9542, CST), anti-RUVBL1 (ab133513, Abcam), anti-CDK1 (#9116, CST), anti-TFG (ab156866, Abcam), and anti-SSBP1 (ab224053, Abcam). After washing the membranes 4 times with TBST, the membranes were incubated with goat anti-rabbit (1:10000, Cell Signaling Technology Inc., MA, USA) or horse anti-mouse (1:10000, Cell Signaling Technology Inc., MA, USA) secondary antibody at RT for 1.5 h. Subsequently, Western blot signals were detected with a Pierce™ ECL Western blot substrate kit (Thermo Fisher Scientific, San Jose, CA, USA). GAPDH was used as the internal reference.

### Cell proliferation assay

Celigo assays and 3-(4,5-dimethylthiazol-2-yl)-2,5-diphenyltetrazolium bromide (MTT) assays were performed to detect cell growth. A Celigo Imaging Cytometer (Nexcelom Bioscience, Lawrence, MA, USA) was used to evaluate cell proliferation. GBC-SD and NOZ cells transfected with sh-GPRIN1 or controls were trypsinized, resuspended, seeded on a 96-well plate (Corning, USA) and then incubated at 37 °C in a humidified atmosphere. Cell proliferation was observed every 24 h, and the cell proliferation curve and doubling time were subsequently determined. At 1, 2, 3, 4, or 5 d post-infection, the cells were incubated with 20 μl of MTT (5 mg/ml; Glenview, Houston, TX, USA). After incubation for 4 h, the supernatant was discarded and replaced with 100 μl of dimethyl sulfoxide (Shanghai Shiyi Chemical Reagent Co., Shanghai, China), and the absorbance was measured at 490 nm with a microplate reader (Tecan Infinite, Männedorf, Switzerland). Optical density (OD) values were then measured at 490 nm with a microplate reader (Tecan Infinite, Switzerland).

### Cell cycle analysis

Cell cycle analysis was performed via fluorescence-activated cell sorting (FACS). The cells (1 × 10^6^) were harvested after reaching > 80% confluence. After trypsinization, the cells were washed twice with precooled DPBS (4 °C, pH = 7.2–7.4). The cells were then resuspended with propidium iodide (Sigma Aldrich, St. Louis, USA) and RNase (Thermo Fisher Scientific, Inc., USA) for 30 min at 37 °C in the dark. Subsequently, FACS analysis was performed with a BD Accuri C6 plus flow cytometer (BD Biosciences, Franklin Lakes, NJ, USA), and the data were analyzed with NovoExpress software.

### Apoptosis assay

Cell apoptosis was detected using an Annexin V-APC apoptosis detection kit (eBioscience, San Diego, CA, USA) after transfection. GBC-SD and NOZ cells transfected with sh-GPRIN1 or empty vector were seeded in 6-well plates. The cells were harvested when the cells reached > 70% confluency. After trypsinization and resuspension, approximately 5 × 10^5^ cells were centrifuged (1300 rpm, 5 min, 4 °C), washed with precooled D-Hanks (4 °C, pH = 7.2–7.4), treated with 1× binding buffer, and recentrifuged (1300 rpm, 3 min, 4 °C). The cells were then harvested and resuspended in 200 μl of 1× binding buffer, and Annexin V-APC was added to the cells. After incubating the cells in the dark for 10 min at RT, the cells were analyzed by flow cytometry.

### Transwell assays

Transwell migration and invasion assays were carried out using 24-well Transwell chambers (3422, Corning, New York, USA). First, 100 μl of serum-free DMEM was added to the upper chambers and incubated at 37 °C in a humidified atmosphere for 1 h. The culture medium was then discarded, and 100 μl of cell suspension (1 × 10^5^ cells/well) was seeded in each well. Then, 600 μl of DMEM containing 30% FCS was added to the lower Transwell chambers. After a 24-h incubation, the chamber was washed twice with PBS, and the cells were fixed with 4% formalin for 30 min and stained with 0.5% crystal violet (Yuanye Bio-Technology Co., Ltd., Shanghai, China) for 3 min. The Transwell chambers were inverted, and the cells were photographed and counted with an inverted optical microscope (XDS-100; Shanghai Caikon Optical Instrument Co., Ltd., Shanghai, China).

### Xenograft mouse model

Four-week-old female BALB/c nude mice were purchased from Shanghai Branch of Beijing Vitonglihua Laboratory Animal Technology Co., Ltd. NOZ cells transfected with sh-GPRIN1 or empty vector were resuspended in PBS at a concentration of 2 × 10^7^ cells/ml and then injected subcutaneously into the right axilla of the nude mice. Tumor xenograft growth was continuously monitored up to 19 d post-inoculation. The tumor volume (V) was calculated as (length × width2) × 0.5. On day 28, the mice were euthanized, and the tumors were excised, weighed, and processed for further analysis.

### Cell lysis, protein digestion and TMT labeling

SDT buffer (4% SDS and 100 mM Tris-HCl, pH 7.6) was added to NOZ cells transfected with sh-CDK1 (negative control shRNA). The lysate was sonicated, centrifuged, and the supernatant was quantified with a BCA protein assay kit (Biyuntian, Jiangsu, China). The protein was mixed with 6X loading buffer and boiled for 5 min. The proteins were separated on SDS–PAGE gel. Protein bands were visualized by Coomassie Blue.

Proteins from each sample were incorporated into SDT buffer. The detergent was discarded with UA buffer (8 M urea and 150 mM Tris-HCl, pH 8.5). Then, 100 μl of iodoacetamide buffer was added, and the samples were incubated for 30 min in the dark. The filters were washed with 100 μl of UA buffer three times, and then 100 μl of 0.1 M triethylammonium bicarbonate (TEAB) buffer. Finally, the protein suspensions were digested with trypsin (Promega) in 40 μl of 0.1 M TEAB buffer at 37 °C overnight.^20^ A 100 μg peptide mixture from each sample was labeled using TMT reagent according to the manufacturer's instructions (Thermo Fisher Scientific, Inc., USA).

### Phosphopeptide enrichment

The peptide mixture was subjected to TiO_2_ phosphopeptide enrichment with a kit. Specifically, the TiO_2_ flow-through and wash fractions were pooled, and the phosphopeptides were then enriched using a High-Select Fe-NTA phosphopeptide enrichment kit (Thermo Fisher Scientific, A32992) according to the manufacturer's instructions. The TiO_2_ eluent and Fe-NTA eluent were vacuum-dried at 45 °C and then dissolved in buffer (0.1% formic acid).

### Easy nLC

After injection, the samples were loaded onto a C18 reversed-phase analytical column (Thermo Fisher Scientific, Acclaim PepMap RSLC 50 μm × 15 cm, nano viper, P/N164943) in buffer A (0.1% formic acid) and separated with a linear gradient of buffer B (80% acetonitrile and 0.1% formic acid) at a flow rate of 300 nl/min. The linear gradient was set as follows: 0 min–5 min, 5% buffer B; 5 min–210 min, 5%–28% B; 210 min–220 min, 28%–38% B; 220 min–230 min, 38%–99% B; and 230 min–240 min, 99% B.

### LC–MS/MS analysis

LC–MS/MS analysis was carried out on an Orbitrap Exploris 480 mass spectrometer (Thermo Fisher Scientific) connected to an Easy nLC (Thermo Fisher Scientific) for 240 min. The mass spectrometer was operated in positive ion mode. MS data acquisition was acquired via a top-10 data-dependent method that dynamically chooses the most abundant precursor ions in a scan range of 350–1800 m/z for HCD fragmentation. Survey scans were acquired with a resolution of 1,20,000 (at m/z 200), an AGC target of 3E6, and a maxIT of 50 ms. MS2 scans were acquired with a resolution of 45,000 (at m/z 200), an AGC target of 2E5, a maxIT of 120 ms, and an isolation width of 2 m/z. Only ions with a charge state between 2 and 6 and a minimum intensity of 2E3 were selected for fragmentation. The dynamic exclusion for the selected ions was 30 s. The normalized collision energy was set to 30 eV.

### MS data analysis

MS/MS raw files were processed using the MASCOT engine (Matrix Science, London, UK; version 2.6) embedded into Proteome Discoverer 2.2 and searched against the UniProt database downloaded from Uniprot_HomoSapiens_20394_20210127. The search parameters consisted of trypsin as the enzyme with a maximum of two missed cleavages permitted. The precursor mass tolerance was set to 10 ppm, and the MS2 tolerance was set to 0.05 Da. Except for TMT labels, carbamidomethyl (C) was set as the fixed modification. The following were selected as variable modifications: acetyl (protein-N-term), oxidation (M), phospho (ST) and phospho (Y). A false discovery rate of 1% for peptides and proteins was enforced by using a reverse database search strategy. Differentially expressed phosphopeptides were identified based on fold change (>1.2) and *p*-value (<0.05) criteria. The MS detection experiments were each repeated three times. Differentially expressed proteins were identified using a 1% FDR threshold (Benjamini–Hochberg correction), a fold-change cutoff > 1.2 (log_2_FC ± 0.26), and a Student's *t*-test *p*-value < 0.05 to ensure statistical stringency.

### Bioinformatics analysis

GO analysis was performed by Blast2GO. Pathway enrichment analysis was performed utilizing the KEGG database. Subcellular locations were predicted by WoLF PSORT software. Predicted kinase-substrate interaction networks were visualized using Cytoscape 3.2.1.

### Statistical analysis

To perform calculations on sample size the online available software G*Power was used. Sample size was determined using as parameters: 1 − β = 0.80, *α* = 5%. All statistical analyses were performed with SPSS 22.0 (IBM SPSS 22.0, Chicago, IL, United States). The differences between the groups were analyzed using Student's *t*-test, chi-square test or Fisher's exact test. Kaplan–Meier survival estimates were performed through Kaplan–Meier analyses, and a Kaplan–Meier curve is shown. A *p-*value < 0.05 was considered to be statistically significant.

## Supplementary Material

Supplementary material Suppl Fig 2.tif

Suppl Fig 1.tif Suppl Fig 1.tif

Supplementary material Suppl

## Data Availability

The data supporting the findings of this study can be obtained from the corresponding author upon request. The datasets used or analyzed during the current study are available from the corresponding author on reasonable request.
