# Supplementary material for: GPRIN1 enhances gallbladder cancer progression and is associated with the CDK1 pathway
Source: Cancer Biol Ther. 2026 Nov 5;27(1):2753819. doi: 10.1080/15384047.2026.2753819 (PMC13757577; doi:10.1080/15384047.2026.2753819)
Supplement: Supplementary file 1 [file KCBT_A_2753819_SM1910.docx]

Suppl.Fig.1 Western blot analysis confirming GPRIN1 and CDK1 knockdown. Western blot showing the protein levels of CDK1 and GPRIN1 in GBC-SD and NOZ cells after infection with control shRNA (shNC), shCDK1, or shGPRIN1 lentivirus. GAPDH was used as a loading control.

Suppl.Fig.2	Dot plots of flow cytometry data. Flow cytometry dot plots showing the distribution of cells for control shRNA (shCtrl) and shRNA targeting GPRIN1 (shGPRIN1) in GBC-SD cells (A) and NOZ cells (B). The axes represent fluorescence intensity in the FITC-A and APC-A channels. Three representative replicates are shown for each condition.
